# The Effectiveness of a Centering Meditation Intervention on College Stress and Mindfulness: A Randomized Controlled Trial

**DOI:** 10.3389/fpsyg.2021.720824

**Published:** 2021-10-21

**Authors:** Stephanie Dorais, Daniel Gutierrez

**Affiliations:** College of William & Mary, Williamsburg, VA, United States

**Keywords:** efficacy, centering meditation, randomized controlled trial, linear mixed model, college health, intervention delivery, online mindfulness

## Abstract

**Background:** Mental health concerns are climbing steadily on college campuses, and universities do not have the staffing and financial resources to address the overwhelming needs of students seeking counseling services. College counselors generally must place students on waitlists or refer them to external resources. Further, during the COVID-19 pandemic, university counselors have been working tirelessly to treat students through online formats. Alternative, online, evidence-based interventions offer college counselors a significant advantage in effectively treating their students. We seek to expand the empirical evidence for mindfulness interventions through online formats for the college population. We registered the study (ISRCTN13587045) at www.isrctn.com.

**Objective:** We examined the effectiveness of a unique online centering meditation and its impact on stress and trait mindfulness in the college population.

**Methods:** Through a randomized controlled trial, the treatment group participated in a 4-week intervention of centering for 10 min each morning and night. We measured stress and mindfulness in both groups through the Perceived Stress Scale and Cognitive and Affective Mindfulness Scale-Revised at baseline, 2 weeks, and 4 weeks.

**Results:** The centering meditation treatment had a statistically significant positive impact on stress and mindfulness compared to a waitlist control group. The meditation group had an average of 64% adherence rate.

**Conclusion:** The study findings indicate that individuals who participate in a 4-week online centering intervention showed improved levels of stress and trait mindfulness over time.

**Clinical Trial Registration:** WHO International Clinical Registry Platform, identifier: ISRCTN13587045.

## The Effectiveness of a Centering Meditation on College Stress and Mindfulness: A Randomized Controlled Trial

Young adults face many stressors upon entering college, from academic rigor to limited financial resources (Saleh et al., 2017). In recent decades, mental health concerns in colleges, such as clinical anxiety, have risen by 100% and are likely to continue growing (Bamber and Morpeth, 2019). Although mental health risks are present in many stages of life, college students encounter a unique risk because they are more likely to engage in problematic coping mechanisms (e.g., substance misuse, disordered eating; Bland et al., 2012; Read et al., 2014). College counselors work tirelessly to combat the rising mental health concerns on campuses. Still, many universities do not have the staffing or financial resources to address the overwhelming needs of students on campus (Xiao et al., 2017). As a result, college counselors often must place students on waitlists or refer them to resources off campus (Iarussi and Shaw, 2016). As the burden on in-person counseling services increases, research has been growing in online therapeutic interventions (Andersson and Titov, 2014; Gutierrez et al., 2020). Further, in light of the COVID-19 pandemic, university counselors have been increasingly treating students through online means (e.g., telehealth, online mental health resources) more than ever before. Even as students return to campus, the necessity for online delivery of mental health resources will likely address the growing requests for mental health resources. More counselors and students have used virtual therapy or online resources than ever before, and online interventions may be a more familiar option for mental health concerns than in previous years.

Even before the COVID-19 pandemic, the college counseling field issued a widespread call for evidence-based complementary and alternative medicine (CAM) to supplement and reduce the demand for in-person counseling (Xiao et al., 2017; Gutierrez et al., 2020). One of the most widely researched and established CAM interventions is meditation (Walsh and Shapiro, 2006; Sedlmeier et al., 2012). Meditation has become a prevalent mental health resource for university students and has already shown effective outcomes through online delivery (e.g., Headspace, Koru Mindfulness; Greeson et al., 2014; Forbes et al., 2018). Because the research on online meditative interventions is still in its early stages, the literature on the efficacy and adherence of online meditative interventions typically centers on non-sectarian, mindfulness-based meditations (Plante et al., 2010; Forbes et al., 2018; Gutierrez et al., 2020). However, research has shown that spirituality potentiates the effectiveness of meditation interventions, thereby increasing the positive effects of meditation (Benson and Stark, 1996; Wachholtz and Pargament, 2005; Wang et al., 2021). For instance, spiritual meditators can sometimes experience enhanced outcomes of their practice based on a phenomenon called the *faith factor* (Benson and Stark, 1996; Fox et al., 2016). Benson and Stark (1996) posited that spirituality had a critical interaction effect with practice that led to a range of positive outcomes. This potentiation is important to consider because college students are expressing more interest in spirituality than previous generations (Longsdorf, 2018). Their growing spiritual interest indicates that a spiritually oriented meditation could better meet the college population's needs and potentiate efficacy and adherence to an online intervention. However, there is little empirical support for online spiritual meditations. For instance, centering prayer is a spiritually oriented meditation popular within contemplative or religious circles (Fox et al., 2015, 2016). However, it had yet to receive experimental examination for its effectiveness. We presented an online centering meditation based on centering prayer and experimentally tested its effectiveness on improving stress and mindfulness during a 4-week study with a college sample.

## Centering Prayer

Centering meditation is based on an early Christian mystic practice called centering prayer developed by the Desert Fathers and Mothers in the third century (Keating, 2002). The focus of centering prayer is interior silence which the early Christian mystics to develop deeper communion with God. Like other concentration meditations that use a symbol for focus (Goleman, 1988), practitioners select a word or symbol they hold sacred (e.g., Shalom, hope) and use it to bring back their mind from distractions. However, unlike many other meditations, centering prayer focuses not on the mind but rather areas of spiritual connection. In completative texts, it is called the “return of the heart” (Pennington, 1980, p. 62) or “attention of the heart” (Bourgeault, 2004, p. 113). The term *centering* stems from the idea that the meditator finds their core center and awakens to awareness of divine presence inside them. The contemplative practice continued for centuries, mainly within the confines of monasteries until Trappist monks Keating, Pennington, and Menninger taught and spread its practice during the 1970's (Keating, 2002). Because of its growing popularity among different populations, we presented the centering meditation as a spiritual practice for individuals from any or no walk of faith. Although based on the Christian tradition, Centering Prayer incorporates pluralistic spirituality similar to other mediations rooted in religion, such as Transcendental meditation (arguably rooted in Hinduism) or Vipassana meditation (rooted in Buddhism; Center for Contemplative Mind in Society, 2020).

Recently, researchers began considering applying centering meditation as an intervention similar to how they use established mindfulness-based practices (Knabb, 2012). Still, there had been no experimental evidence of its efficacy (Fox et al., 2016). Several pilot studies offer preliminary empirical support for its positive psychosocial effects (Ferguson et al., 2010; Fox et al., 2016). Ferguson saw statistically significant reductions in stress with a large effect size (*d* = 1.40) after 15 church parishioners engaged in a 2-h centering prayer intervention. In another pilot study, Fox et al. (2016) found statistically significant correlations between centering prayer and improved outcomes, including depression, anxiety, stress, spiritual transcendence, and mindfulness among a group of counseling practitioners participating in centering prayer workshops over several months. However, none of these studies measured its efficacy through experimental design or with online delivery. Nevertheless, their findings provided the basis for our online randomized controlled trial to measure the efficacy of a centering meditation on stress and mindfulness.

## Millennial Spirituality and Adherence

Over recent decades, young adults in the U.S. have been increasingly gravitating to spiritual approaches in wellness routines (Longsdorf, 2018). A decade ago, nine out of 10 college students express interest in spirituality (Astin and Astin, 2010). Researchers have noticed this trend continues to increase among young adults, giving a name to a revival called *millennial spirituality* (Longsdorf, 2018). From a counseling perspective, addressing clients' spiritual wellness is critical to multicultural competence (Fox et al., 2015). While previous experimental trials have provided outcome research for various online meditation interventions (e.g., Koru, Headspace), this study offers new information for spiritual meditation with online delivery. Empirical support for spiritual meditations like a centering meditation is sparse, especially with a population that does not necessarily affiliate with any one religion (Gutierrez et al., 2016). Thus far, Gutierrez et al. (2016) demonstrated the effectiveness of a spiritually-based meditation called Jyoti in improving stress among counseling graduate students. Similarly, Knabb and Vazquez (2018) empirically supported the effect of the Jesus Prayer meditation in decreasing stress in a sample of Christian college students. However, there is still much to learn about the effectiveness of different spiritual meditations with various populations. Further, given the incoming wave of *millennial spirituality*, the findings could have unique implications on the current generation of college students. Additionally, effectiveness depends on practitioners adhering to the practice for more extended periods.

The effectiveness of meditation correlate to adherence (Forbes et al., 2018). Based on previous studies, it is difficult for college students to maintain adherence to online meditations. For instance, in a 10-session meditation study, 53% of college students rarely completed each meditation in full (Forbes et al., 2018). Based on various mindfulness meditation studies, adherence rates generally range from 23 to 53% (Cavanagh et al., 2013; Howells et al., 2014; Forbes et al., 2018; Flett et al., 2020). It is sometimes complicated for researchers to assess the efficacy of long-term daily interventions like meditation because of low adherence (Flett et al., 2020). While long-term meditation practice takes discipline, researchers aim to examine what could bolster adherence rates. Because each of the meditations is non-sectarian, there is a research gap on the adherence rates of spiritual meditations. Given the penchant for spirituality in the current college population, it could be that a spiritual meditation could potentially lead to greater adherence in online meditation. The following research question guided our study to address the unknowns in spiritual, online meditation: Is there a significant difference in stress and mindfulness between students who participate in a bi-daily centering meditation and a waitlist control group?

## Method

### Study Design

This experimental study was a 4-week randomized controlled trial that compares the effects of a centering meditation group to a waitlist control group on stress and mindfulness. The length of the study (4 weeks) and frequency of meditation (twice a day) is based on the procedures of related empirical studies (Offidani et al., 2017; Knabb et al., 2020). The study protocol includes Intention-to-Treat (ITT) analysis to reduce any biased effect of the intervention (Gupta, 2011). Adhering to Consolidated Standards of Reporting Trials (CONSORT) guidelines in this study, the researchers reported all participants who did not follow through with the intervention. The main analysis includes linear mixed modeling to estimate the fixed and random effects of the intervention.

### Participants

**Figure 1:**
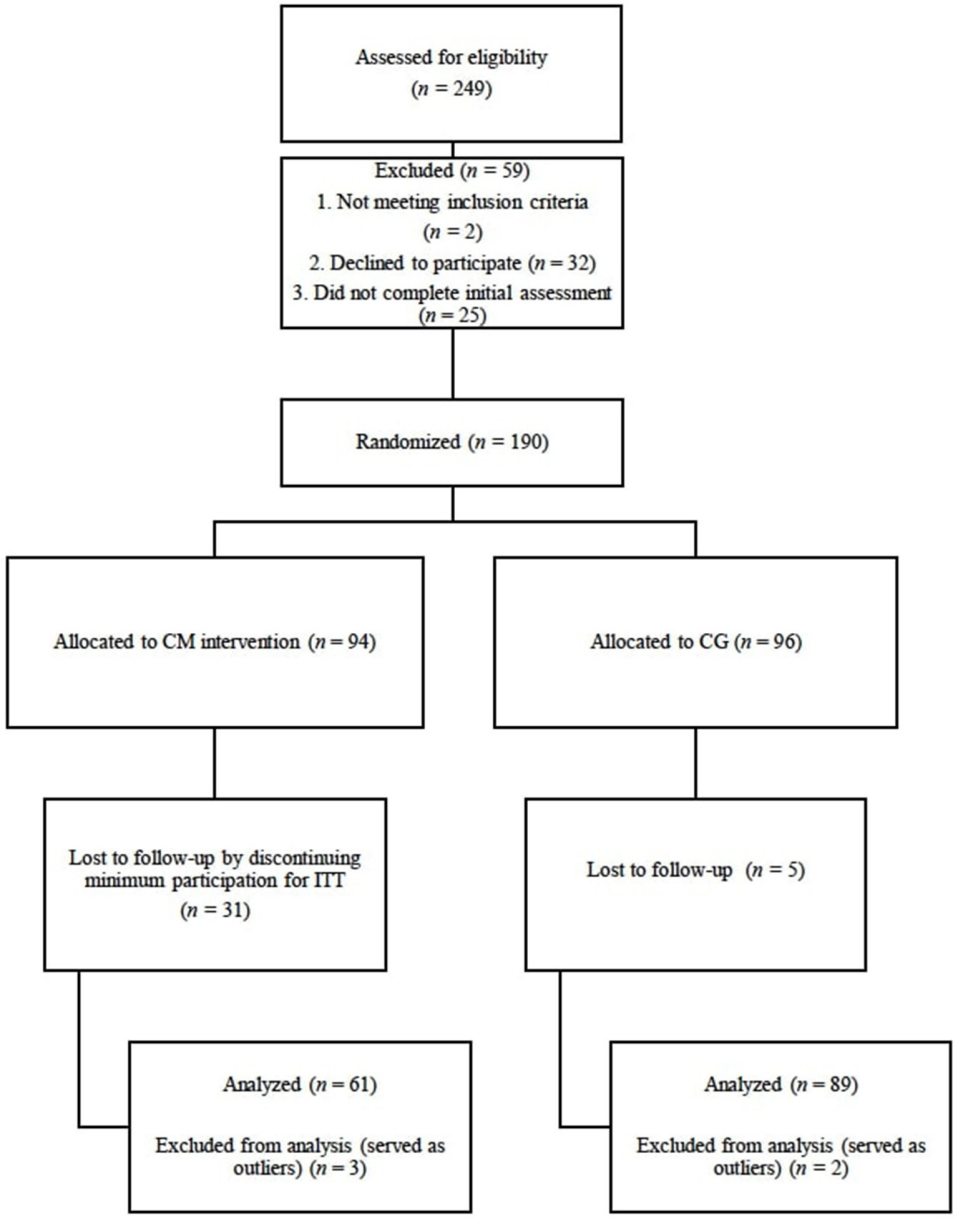
Participant CONSORT flow diagram through the study. CM, centering meditation; CG, comparison group.

The targeted sample size was based on linear mixed modeling recommendations of sample sizes of at least 100 participants (Curran et al., 2010). Inclusion criteria consisted of (a) age above 18 years and (b) part-time or full-time enrollment in an undergraduate or graduate program. Anticipating over a 50% attrition rate, we recruited 249 participants in our initial sample. The CONSORT flow diagram in Figure 1 presents details on the enrollment procedure. Only 190 participants met all the requirements to enroll in the study formally. Out of the 190 participants, the final sample excludes 35 participants for not completing the protocol based on Intention-to-Treat standards (McCoy, 2017) and five participants as outliers based on high influences statistics. Participants were able to receive compensation in installments to a total of $35, depending on adherence to the study. Table 1 presents a detailed list of the demographic information of participants.

**Table 1:** Summary of participant descriptive statistics.

	**Eligible sample participants at baseline protocol** **(*n* = 190)**	**Final sample of participants in data analysis** **(*n =* 150)**	**Final treatment group** **(*n =* 61)**	**Final control group** **(*n =* 89)**
Age, mean, SD	27.03 (7.61)	27.10 (7.38)	26.66 (5.96)	27.45 (8.70)
**Gender**, ***n***				
Male	26 (14%)	19 (13%)	5 (8%)	14 (16%)
Female	157 (83%)	126 (84%)	53 (87%)	73 (82%)
Non-binary	4 (2%)	3 (2%)	1 (2%)	2 (2%)
Prefer not to answer	3 (2%)	2 (1%)	2 (3%)	0 (0%)
**Race**				
White	125 (66%)	97 (65%)	39 (64%)	58 (65%)
Multiracial	21 (11%)	19 (13%)	10 (16%)	9 (10%)
Hispanic/Latinx	20 (11%)	16 (11%)	2 (3%)	14 (16%)
Black/African American	16 (8%)	12 (8%)	6 (10%)	6 (7%)
Asian	4 (2%)	3 (2%)	2 (3%)	1 (1%)
Other/Prefer not to answer	4 (2%)	2 (2%)	2 (3%)	1(1%)
**Level of Education**				
Undergraduate	26 (14%)	21 (14%)	9 (15%)	12 (13%)
Graduate	164 (86%)	129 (86%)	52 (85%)	77 (87%)
**Spiritual/Religious Identity**				
Spiritual	68 (36%)	50 (33%)	19 (31%)	31 (35%)
Spiritual and religious	32 (17%)	27 (18%)	12 (20%)	15 (17%)
Religious	25 (13%)	18 (12%)	6 (10%)	12 (13%)
None	53 (28%)	45 (30%)	17 (28%)	28 (31%)
Other/prefer not to answer	12 (6%)	10 (7%)	7 (6%)	3 (3%)

### Procedure

Upon receiving approval from our institutional review board, the researchers contacted various institutions and universities (e.g., community colleges, universities, and student organizations) to recruit our target population of college students. After receiving permission from professors or organizational leaders, the researchers distributed information about the study to college students through email and listservs. The emailed information included an online video and written transcript of the first author explaining the study's purpose, procedure, and compensation. In the end, individuals who wished to enroll could follow a web link to enroll in the study *via* Qualtrics. Before beginning the protocol, participants gave informed consent and passed the inclusion criteria screener. Participants were aware their participation was voluntary and could withdraw from the study at any point in time. Upon enrollment, all participants completed a baseline protocol on Qualtrics that included the Perceived Stress Scale (PSS; Cohen et al., 1994) and Cognitive and Affective Mindfulness Scale-Revised (CAMS-R; Feldman et al., 2007). After completing the baseline protocol, a randomized generator Qualtrics randomly separated participants into either a treatment or control group. All participant information regarding their assignment was blinded to both the researchers and the participants. This study stems from a larger project that assessed for multiple mental wellness concerns. Thus, the researchers were able to present this study without mention of meditation, which helped with the blinding aspect. We presented the study as research on coping and goal-attainment among students. Thus, participants in the control group could believe they were receiving assessments based on their stress levels and coping over the semester. To further control for confounding variables such as control group participants practicing meditation, the researchers asked all participants to list any contemplative practices that they engaged in regularly.

Participants actively engaged in the study during September and October of 2020. All participants received compensation weekly. They received installments of $5 depending on the number of times they turned in their daily assessments and whether or not they completed the larger assessment batteries. To monitor adherence, Qualtrics sent participants reminders with links to complete questionnaires at specified times. Completing the brief morning or evening assessment served as verification of adherence. After the study was completed, the waitlist control group received access to the intervention as well.

### Treatment

Once receiving their blinded assignment to the treatment group, participants received an email to watch an online training video for centering meditation. In the video, a counselor formally trained in centering prayer meditation narrated the guided meditation. The guided meditation video explained the concepts of spiritual or sacred words to make them applicable to those who did not identify themselves as spiritual. The video offered explanations of words that are considered meaningful or important such as hope or joy. In addition to the guided meditation video, participants could follow written instructions based on Keating's guidelines to centering prayer (Keating, 2002).

1. Select a word or symbol that you consider spiritually grounding for you (e.g., a name for God or spiritual concepts like Shalom, hope, or joy) and represents your intention to connect with what is spiritual to you.
2. At the beginning of the meditation, sit down comfortably with your eyes closed (preferably away from external distractions) and silently introduce your sacred word or symbol.
3. After you do this, you'll probably notice distracting thoughts emerging or mind wandering. That's okay. When you notice yourself becoming distracted, just ever-so-gently reintroduce your sacred word or symbol.
4. At the end of your meditation, just sit and rest in silence or say a prayer for a few moments.

Participants in the treatment group received a reminder email every morning and evening to practice their meditation. The email included a link to the guided meditation video, the written guidelines, a 10-min timer, a sliding scale measure of the length of their practice, and a brief assessment which included open-ended questions of any concerns during their meditation. Participants in the control group received the same bi-daily assessment without any meditation materials. The guidelines asked them simply to consider their goals and their motivation in completing them for that time of the day. Participants in the treatment and control groups completed the same protocol of stress and mindfulness at the beginning (T_1_; Day 1), midpoint (T_2_, Day 14), and end of the study (T_3_, Day 28). Consult Table 2 for a further description of participant scores.

**Table 2:** Univariate descriptive statistics for the perceived stress scale (PSS) and cognitive and affective mindfulness scale-revised (CAMS-R) by group.

	**Stress**			**Mindfulness**		
	**T_**1**_**	**T_**2**_**	**T_**3**_**	**T_**1**_**	**T_**2**_**	**T_**3**_**
**Treatment Group (*****n =*** **61)**						
Mean	20.07	18.95	17.52	31.07	31.93	32.84
Standard deviation	4.74	5.26	5.01	5.19	4.63	4.98
**Comparison Group (*****n =*** **89)**						
Mean	20.03	19.14	19.49	31.30	31.60	31.61
Standard deviation	5.76	6.45	5.66	5.04	5.50	5.32

*Time refers to three time points T_1_= Week 0, T_2_ = Week 2, T_3_ = Week 4*.

### Measures

#### Demographic Questionnaire

At the beginning of the study, we asked all participants to report on age, gender, spirituality/religious affiliation, and academic level (e.g., undergraduate). Although it was not required, participants had the opportunity to include a specific religion or denomination to which they belonged (e.g., Jewish, Presbyterian). We also asked participants to describe any contemplative practices (e.g., yoga, meditation) that they engaged in.

#### Stress

To measure the effect of the centering meditation on stress, we administered the Perceived Stress Scale (PSS; Cohen et al., 1994). Because we built this study on Lazarus and Folkman's seminal transactional stress theory (Lazarus and Folkman, 1984), we chose this scale specifically because it differs from objective assessments of stress and measures one's perception or cognitive appraisal of stress. In other words, the items ask respondents to rate their thoughts and feelings (e.g., feelings of irritability, thoughts of not being able to cope) over the last month. In addition, meditation practice associates with improved scores of the PSS (Lane et al., 2007; Chu, 2010; Gutierrez et al., 2016). Participants respond to items on a 5-point Likert scale ranging from Never to Very Often. We summed the scores and used the total scores as the outcome variable in the study.

Cohen et al. (1994) developed several versions of the scale, including a 14-, 10-, and 6-, 4-item scale (Lee, 2012). The two larger scales have a 2-factor solution, while the smaller scales are unidimensional. However, we selected the 10-item scale because it has the strongest psychometric properties across several populations (Lee, 2012). In a sample of adults with chronically ill children, the PSS-10 demonstrated acceptable test-retest reliability (*r* = 0.77) over 2 weeks (Remor, 2006). In 11 studies with adult samples mainly comprised of college students, it provided evidence of internal consistency through Cronbach's alpha (α = 0.74–0.91; Lee, 2012). Its 2-factor solution continually displays a good fit through exploratory factor analysis and confirmatory factor analysis. It demonstrated convergent validity (*r* = 0.73) with *the* State-Trait Anxiety Inventory for Adults (STAI-AD; Spielberger, 1983) as well as discriminant validity through no correlation with the Adult Overt Aggregation Scale (OA; Roberti et al., 2006). The sample in the present study provided evidence of internal consistency at each time point in the study α_T1_ = 0.84, α_T2_ = 0.89, α_T3_ = 0.84).

#### Mindfulness

To assess levels of mindfulness among participants, we administered the 12-item Cognitive and Affective Mindfulness Scale-Revised (CAMS-R), which Feldman et al. (2007) adapted from the original 18-item CAMS. Trait mindfulness refers to a personality disposition in which a person demonstrates an average expected level of mindfulness over time. The counter phenomenon is *state mindfulness*, which reflects a state of mind that could shift dramatically from one point to the next. We selected a trait measurement because research suggests that meditation interventions will significantly change levels of trait mindfulness over time (Kiken et al., 2015). In other words, individuals increase their states of mindfulness during meditation, but it regresses after meditation. However, as they meditate daily over time, state mindfulness will stabilize at higher levels, thus increasing their trait mindfulness. Therefore, we selected the CAMS-R since we plan to measure mindfulness over time and because it operationalizes mindfulness as a “trainable” trait (Gawrysiak et al., 2018), as it measures traits of mindfulness that could change with an intervention.

The CAMS-R aims to measure everyday aspects of mindfulness that apply whether respondents purposefully practice mindfulness or not, making it applicable to a wide variety of participants. Participants respond to the items on a 4-point Likert scale (1 = “Rarely/ Not at all”, 4 = “Almost always”). We scored the items based on total scores (Feldman et al., 2007). The CAMS-R measures mindfulness as a multidimensional construct, assessing aspects of (a) Attention, (b) Awareness, (c) Acceptance, and (d) Focus. Researchers have validated this 4-factor structure across several international populations through confirmatory factor analysis (Feldman et al., 2007). Further, the CAMS-R continuously demonstrates convergent validity (*r* = 0.51, 0.66) with related dispositional mindfulness scales (Feldman et al., 2007). Further, it demonstrated evidence of discriminant validity through its negative correlation (*r* = −0.30) with rumination [as measured by the Response Style Questionnaire (RSQ; Nolen-Hoeksema and Morrow, 1991)]. Although the four subscales demonstrate evidence of construct validity, the developers recommend using a total score because the internal consistency is higher (Feldman et al., 2007). Thus, we use a total score in our analysis. In addition, the sample from the study demonstrated evidence of internal consistency through Cronbach's alpha at each time point in the study (α_T1_ = 0.79, α_T2_ = 0.80, α_T3_ = 0.82).

### Preliminary Analysis

After 4 weeks of data collection, we uploaded the data from 155 participants onto Statistical Analysis System (SAS). Influence statistics identified five participants as outliers. With a final sample of 150 participants, the data of stress and mindfulness both followed normal distributions based on the Shapiro-Wilks test, *p* > 0.05. Before addressing the research question, we conducted an unconditional growth model to determine if the repeated measures data was appropriate for linear mixed modeling. Table 3 presents the outcome of the unconditional growth models. A recommended first step in linear mixed modeling, the unconditional growth models allowed us to calculate the intraclass correlation (ICC) for both stress (ICC = 72.88%) and mindfulness (ICC = 84.34%). The ICC reflects variation in the outcome between individuals (Grace-Martin, 2013), which means within-person variance over time over 20% of the variance. Thus, it is important to examine the within-person variance through random effects parameters in linear mixed modeling.

**Table 3:** Parameter estimates for the growth curve models on outcomes of stress and mindfulness.

	**Stress**		**Mindfulness**	
	**Unconditional growth model**	**Conditional growth model**	**Unconditional growth model**	**Conditional growth model**
**Fixed Effects** ***(SE)***				
Intercept	19.97^***^ (0.44)	19.83^***^ (0.58)	31.22^***^ (0.42)	31.33 *(0.54)*
Time^a^	−0.59^**^ (0.19)	−0.13 *(0.24)*	0.40^*^ (0.17)	0.01 *(0.22)*
Group^b^		0.34 *(0.91)*		−0.28 *(0.85)*
Time*Group		−1.12^**^ (0.38)		0.94^**^ (0.35)
**Random Effects** ***(SE)***				
UN(1,1) Intercept τ^2^ (Between-person)	22.44^***^ (3.53)	22.51^***^ (3.55)	22.57^***^ (3.06)	22.66^***^ (3.09)
UN(2,2) Time	1.04 *(0.82)*	0.71 *(0.80)*	2.21^***^ (0.60)	2.00^***^ (0.58)
Residual σ^2^ (Within-person)	8.35^***^ (1.02)	8.40^***^ (1.02)	4.19^***^ (0.52)	4.19^***^ (0.52)
ICC	72.88%		84.34%	

The estimates for Time, Group, and Time*Group reflect standardized beta weights.

a *Time refers to three time points T_1_ = Week 0, T_2_ = Week 2, T_3_ = Week 4. The slope indicates the change in stress or mindfulness per 14-day time period*.

b *Group refers to the treatment group and control group*.

* *p < 0.05*.

** *p < 0.01*.

*** *p < 0.001*.

We applied linear mixed modeling through PROC MIXED for all analyses. We chose the mixed model over an ANOVA because we wanted to account for the random effects over time (Grace-Martin, 2013). The main effects included group (treatment vs. control), time (T_1_, T_2_, T_3_), and group-over-time interaction. The interaction effect uses time to compare the groups at each time point as well. The model estimated the random effects of time through an unstructured covariance structure. As mentioned previously, we used ITT analysis and thus used the last recorded observation of any participants who did not follow the protocol in full (Gupta, 2011). To measure effect size, we computed Cohen's *d* (1988) as the mean difference between pre- and post-test scores over their baseline pooled standard deviation. We report on the effect size and demonstrate the interpretation based on Cohen (1988) and Lovakov and Agadullina (2021).

## Results

### Stress Response to Centering Meditation

After a 4-week treatment of centering meditation, the interaction between group and time was statistically significant in explaining the trajectory of stress (β = −1.12, *SE* = 0.38, *df* = 140, *p* < 0.01, *CI*_95_ = −1.87, −0.36). The fixed effect of −1.12 reflects the difference in slope between the treatment and control groups (Bolger and Laurenceau, 2013). As the time^*^group interaction effect in Table 3 demonstrates, the treatment group over time did yield a significant difference in stress compared to the control group. The treatment group generated a medium, within-group effect size of *d* = 0.52 (Cohen, 1988; Lovakov and Agadullina, 2021). The means in stress grew slightly in the control group, but the effect was smaller than Cohen's cut-off for a small effect (*d* = 0.10). Figure 1 presents a graph of the group-specific effects on stress over time. As Table 3 indicates, time and group each had nonsignificant effects on stress. It is expected for group to not have a statistically significant effect in a 3-time point study. This is because we purposefully randomized assignment to the treatment and control group, thus their scores are not expected to differ at baseline. They differ by the third time point, but time would have to be included in that interaction to see a statistically significant difference between groups.

### Mindfulness Response to Centering Meditation

**Figure 2:**
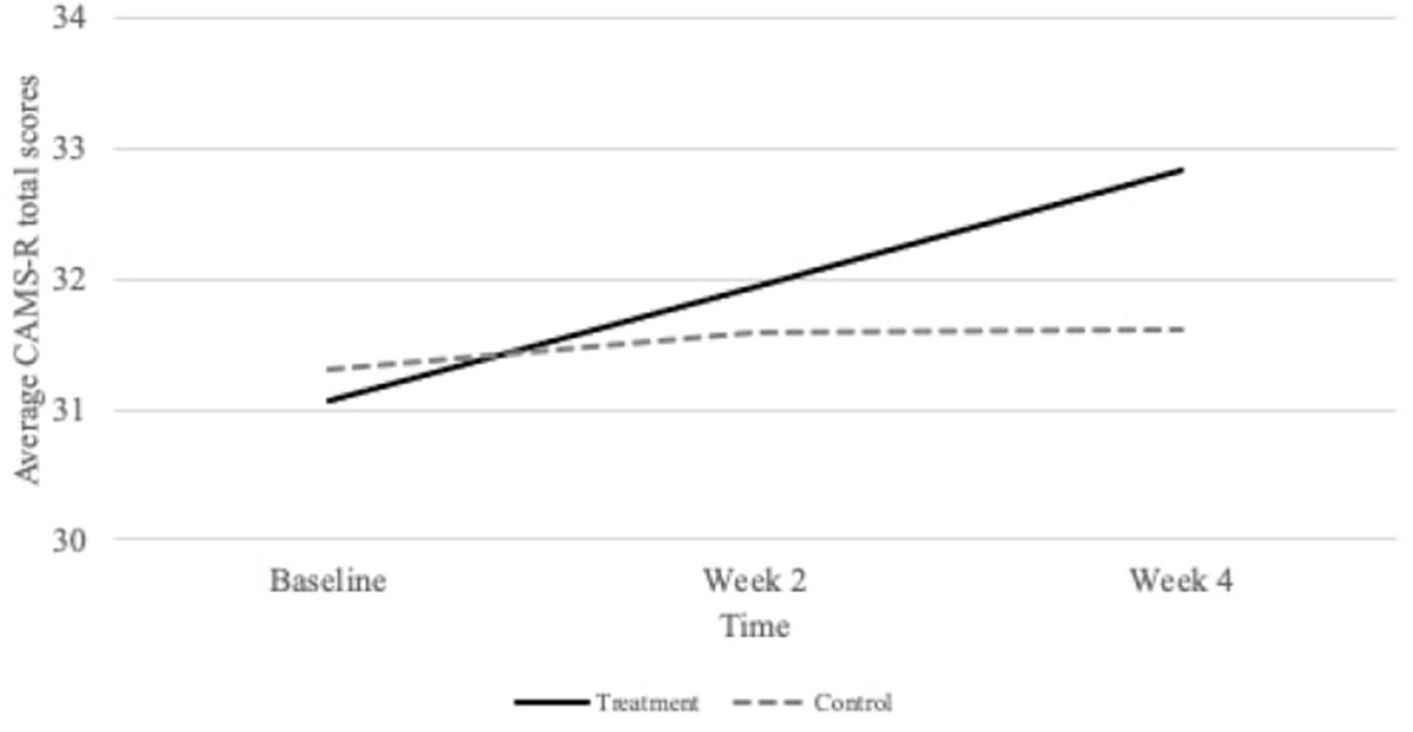
Group specific changes in mindfulness as measured by the Cognitive and Affective Mindfulness Scale-Revised (CAMS-R) at baseline, 2 weeks, and 4 weeks.

After a 4-week treatment of centering meditation, the interaction between group and time was statistically significant in explaining the trajectory of mindfulness (β = 0.94, *SE* = 0.35, *df* = 138, *p* < 0.01, *CI*_95_ = 0.26, 1.62). The fixed effect indicates that the mean slope of the treatment group was 0.94 units greater than the control group's mean slope (Bolger and Laurenceau, 2013). In other words, CAMS-R scores from the treatment group increased by 0.94 units more than the control group every 2 weeks. As Table 3 indicates, similar to the outcome of stress, the covariates of time and group alone had non-significant effects on mindfulness. Once again, we would not expect significant group differences at T_1_ because we randomly separated participants between groups. Time was also not a statistically significant factor. Without accounting for groups, there is little theoretical explanation for why mindfulness would grow statistically significantly just over time alone. However, the interaction of the treatment group and time did yield a significant effect on increasing mindfulness. Figure 2 presents a graph of the group-specific effects on stress over time.

The treatment group generated a within-group effect size of *d* = 0.35, a small-to-medium effect (Cohen, 1988; Lovakov and Agadullina, 2021) based on the study's sample size and experimental design. The change in means in the control group was negligent (*d* = 0.06). Another method to estimate effect compares the unconditional growth model results with the treatment growth model. Unlike the stress model, the linear mixed model of meditation treatment did not reduce random variation in mindfulness. This means that other measures such as Repeated Measures ANOVA would be appropriate for measuring longitudinal change with this sample.

### Adherence

**Figure 3:**
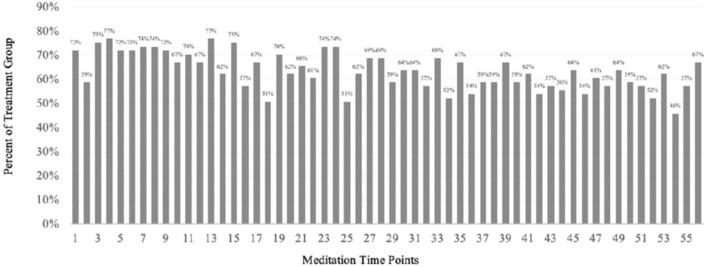
Percentage of adherence in treatment group at each of the 56 meditation sessions in the 4-week study.

The average participant in the treatment group participated in 36 out of the 56 meditation sessions over 4 weeks, yielding an adherence rate of 64%. We also computed the adherence rate as the percentage of all 61 participants who engaged in each bidaily meditation session. Figure 3 presents the percentage of participants practicing per meditation session. The average adherence rate is still 64% with this computation. The average adherence rate was 66% in the morning and 61% in the evening. The adherence rates ranged between 46 and 77% in the study.

## Discussion

Our study stems from the first randomized controlled trial on the effectiveness of centering meditation. The findings indicate that centering meditation improves stress and mindfulness with statistical significance over time. Further, the linear mixed modeling approach demonstrated that the centering meditation significantly reduced stress between groups and reduced the variance of stress within a participant. It is important to explore how the effects of centering meditation differ or align with meditations in its categories. The literature has three common categories: devotional, focused, or mindfulness meditations (Gutierrez et al., 2015). Centering is in a way both devotional and focused. It is devotional because of its spiritual roots and purpose, but the function is the focus on a mantra. The centering meditation is unique because it is a distinctly spiritual meditation. Unlike many other mindfulness practices stripped of their spiritual roots (Plante et al., 2010), centering meditation directly draws from the meditator's spirituality. This study adds to the research on the small pool of empirical evidence for spiritual meditations for college students. Our findings align with Knabb and Vazquez's (2018) findings. In a 2-week randomized controlled trial, Knabb and Vazquez (2018) demonstrated that the daily, devotional meditation of the Jesus Prayer significantly reduced stress with a large effect in a sample of Christian university students. Their findings support the efficacy of spiritual meditations, but the generalizability is narrow because of the Christian religious orientation of participants. As Table 1 indicates, the participants in our study came from various walks of faith. One out of three participants did not even affiliate with spirituality, but they still benefited and adhered to spiritual meditation.

Centering meditation is also similar to focused or concentration meditations like Transcendental Meditation, which has become renowned in the previous research for reducing stress and improving mental wellness (Gutierrez et al., 2015; Dorais and Gutierrez, 2021). Even though it is not considered a mindfulness meditation, meditators engaging in centering meditation appears to induce similar neurological activity to subjects engaging in other established mindfulness practices (Newberg et al., 2003; Gutierrez et al., 2015). Although we did not assess neurological or physiological activity in our study, we saw changes in mindfulness and stress that often correspond to reduced heart rate, decreased cortisol, and changed neural pathways seen in the literature (Heckenberg et al., 2018; Kwak et al., 2019). For instance, in their randomized controlled trial, Kwak et al. (2019) saw similar increases in scores of the CAMS self-report measure we used, but they also observed improved resting-state functional connectivity (rsFC). Thus, it is possible similar neural mechanisms led to the changes we saw in our study.

Our findings also align with those of other randomized controlled trial studies on college meditation. It demonstrates a similar reduction in stress over time compared to in-person group meditations such as Mindfulness-Based Stress Reduction (MBSR; Kabat-Zinn and Thich, 2013) and the Easwaran's Eight-Point Program (EPP; Easwaran, 1991) among college students (Oman et al., 2008). The similarity supports how online interventions may potentially yield similar results as in-person interventions, and our findings provide evidence from which future studies could benefit (Andersson and Titov, 2014). Our findings also support the research literature on the efficacy of online meditations in improving stress and mindfulness (Huberty et al., 2019). In a randomized controlled trial with college students, Huberty et al. (2019) demonstrated the effectiveness of the “calm” app in improving stress and mindfulness over time. In another randomized controlled trial (Cavanagh et al., 2013), college students enrolled in “Learning Mindfulness Online” demonstrated improved perceived stress and mindfulness. Flett et al. (2020) found slight improvements in college distress after applying the Headspace app as the intervention in a randomized controlled trial, but the researchers indicate the weak results were due to low adherence (as they found greater improvements among students who participated more in the meditation).

**Figure 4:**
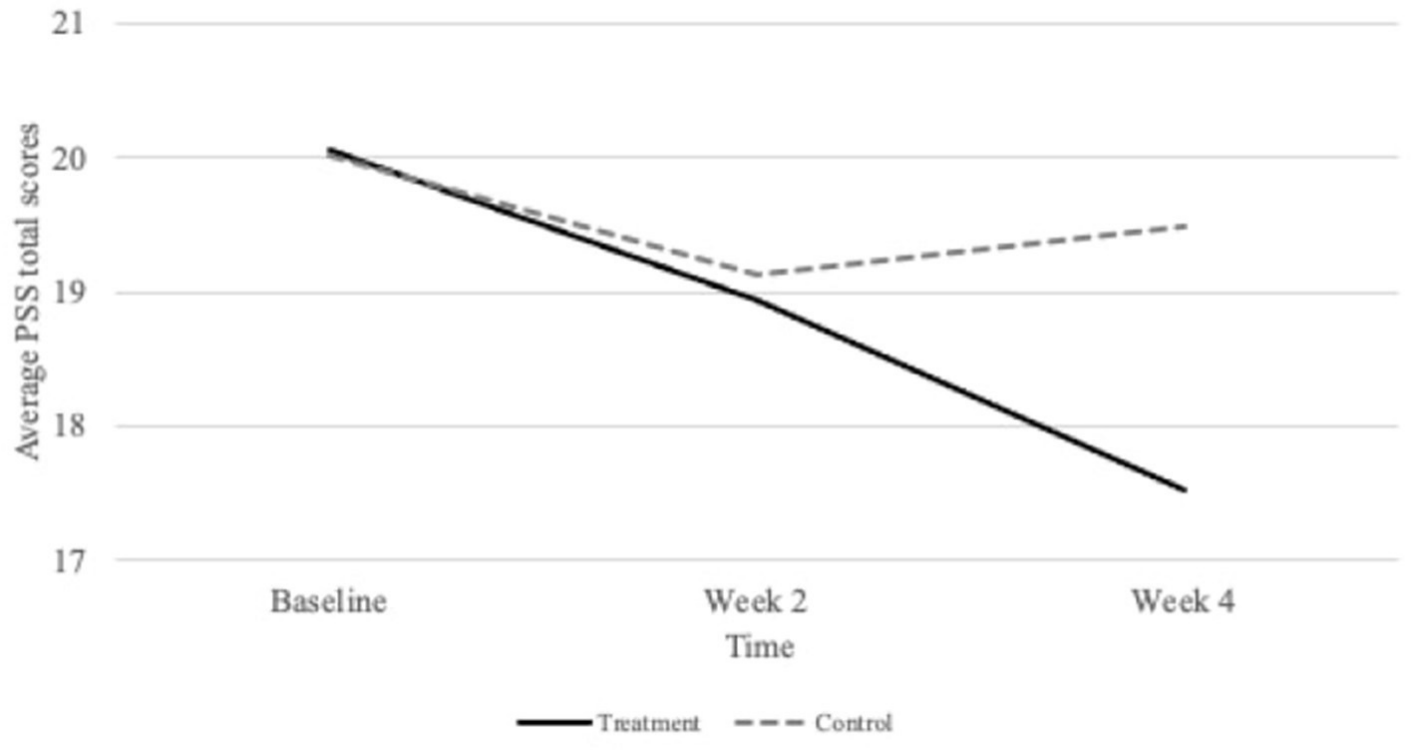
Group specific changes in stress as measured by the Perceived Stress Scale (PSS) at baseline, 2 weeks, and 4 weeks.

As Flett et al. (2020) mentioned in their study, adherence to meditation can help improve the efficacy of the practice (Forbes et al., 2018). Further, it is especially critical in our study as we present centering meditation as a viable CAM intervention to counseling sessions. Comparing adherence rates between studies is complex because different meditation studies implement different numbers of required sessions. For instance, Forbes et al. (2018) conducted a study on adherence to a 10-session mindfulness meditation. In a sample of college students (*n* = 169), 53% of participants practiced all 10 meditation sessions. Our sample consisted of 56 meditation sessions, and the highest “full adherence” rate was three participants who practiced 55 of the 56 meditations. Nevertheless, overall an average of 64% of the participants practiced the meditation. Further, as Figure 4 indicates, the lowest adherence rate at any session was 44%. Researchers calculate adherence similar to attrition in some studies by comparing pre- and post- intervention (Cavanagh et al., 2013). Based on ITT procedure (McCoy, 2017), we retained all participants who completed more than one assessment. Thus, we retained 61 participants of the 94 participants in the treatment. From this calculation, we had a 65% adherence rate.

### Limitations

Because the purpose of the study was to examine the effectiveness of an online centering meditation, it is essential to simultaneously consider the limitations of any study in which the researchers could not personally monitor procedures. One of the limitations of home-based studies is that participants reported their treatment doses (length of bi-daily meditation) as we could not record it ourselves. Dosage levels are critical in intervention research (Shadish et al., 2002) because it directly connects the intervention to measured outcomes of stress and mindfulness. To mitigate this limitation, each participant had to include their doses of meditation (minutes spent meditating) to submit their bi-daily surveys. We used a force-response feature on Qualtrics to ensure that they reported their dosage at each survey administration. Further, to mitigate this concern, we clearly stated that if they did not practice their meditation, all they had to do was to report it in the daily assessment sent to them. We highlighted and ensured that we compensated participants based on their submitting the daily assessment, regardless of whether they practiced their meditation. With this compensation procedure, we reduced reasons to misreport treatment dosage. A second limitation concerns attrition. Since participation in this study was voluntary, participants were free to withdraw from the study at any time of their choice. When the study began, the treatment group and control group each consisted of 95 participants. Participants had to complete at least one follow-up procedure based on ITT procedure to remain in the study (McCoy, 2017). After enrolling in the study, the control group decreased by 6% and the treatment group by 35%. We handled missing data through different approaches. If a participant completed an assessment, there was no missing data on each instrument since we used a forced-response feature on Qualtrics. However, not all participants completed every assessment at T_1_, T_2_, and T_3_. The PROC MIXED analysis we used automatically accounts for missing observations in a data set through simulation procedures (Johnson, 1999). Lastly, the generalizability of our study is another limitation. Most of our participants were white females, but the demographic breakdown is also typical of studies on mindfulness and wellness with the college and graduate population (Baldwin et al., 2017). A final limitation relates to counseling practice. Although this provides empirical support for a type of spiritual integration, a limitation could be that it would not be conducive to treatment for individuals who do not desire spiritual integration in practice.

### Recommendations for Future Research

One purpose of the noted limitations is to improve future research that aims to build upon this study. Based on both the findings and limitations, we note several recommendations for future research. The first recommendation is to replicate the study to measure another outcome beyond stress or mindfulness. Based on the results, centering meditation improved stress and mindfulness similarly to other established mindfulness programs (e.g., MBSR; Kabat-Zinn and Thich, 2013). The literature shows that many of these meditations confer many different desirable outcomes such as improved sleep, chronic pain, mood, and relational satisfaction (Sedlmeier et al., 2012; van der Velden et al., 2015). Although it was beyond the scope of this study, the findings provide a basis to measure the outcomes of such desired outcomes based on centering meditation. The fields of contemplative science and positive psychology already give a theoretical basis to test the efficacy of centering meditation on these constructs, and previous literature supports it as well.

A second recommendation would be to examine the underlying mechanisms of centering meditation. The underlying, theoretical factors through which an intervention leads to therapeutic outcomes are known as *mechanisms of change* (Petrik and Cronin, 2014). If mental health professionals begin using centering meditation as an evidence-based CAM intervention for college students, it is vital to have a deeper understanding of its mechanisms (Petrik and Cronin, 2014). Contemplative research moves beyond outcome studies of meditation and toward mechanistic studies of *how* they work (van der Velden et al., 2015). Analyses such as longitudinal within-subject mediation could determine what constructs (e.g., spiritual transcendence, hope) lead to the positive effects of centering meditation on stress and mindfulness (Bolger and Laurenceau, 2013). Preliminary research in this area has presented favorable outcomes (see Dorais and Gutierrez, 2021). The findings from this research could expand the use of centering meditation as a counseling intervention.

## Conclusion

The study's primary purpose was to test if centering meditation could decrease stress and increase mindfulness among college students. While meditation is a widely accepted agent of change for mental health outcomes (Sedlmeier et al., 2012), centering meditation's effectiveness had limited to no empirical support especially with a college population (Fox et al., 2016). This study provides the college population with an evidence-based practice for improving stress and mindfulness, which individuals can use outside of counseling sessions. Records of the psychological benefits of this lesser-known spiritual practice existed over centuries (Keating, 2002). As centering meditation shows a resurgence in the wellness practices today (Fox et al., 2016), this study now provides the first empirical evidence of its mental health benefits and hopefully paves a path for further research on the effectiveness of centering meditation.

## Data Availability Statement

The raw data supporting the conclusions of this article will be made available by the authors, without undue reservation.

## Ethics Statement

The studies involving human participants were reviewed and approved by William & Mary Institutional Review Board. The patients/participants provided their written informed consent to participate in this study.

## Author Contributions

Both authors listed have made a substantial, direct and intellectual contribution to the work, and approved it for publication.

## Conflict of Interest

The authors declare that the research was conducted in the absence of any commercial or financial relationships that could be construed as a potential conflict of interest.

## Publisher's Note

All claims expressed in this article are solely those of the authors and do not necessarily represent those of their affiliated organizations, or those of the publisher, the editors and the reviewers. Any product that may be evaluated in this article, or claim that may be made by its manufacturer, is not guaranteed or endorsed by the publisher.
